# Fracture-pattern-related therapy concepts in distal humeral fractures

**DOI:** 10.1007/s11678-018-0442-8

**Published:** 2018-02-15

**Authors:** Rony-Orijit Dey Hazra, Helmut Lill, Gunnar Jensen, Julia Imrecke, Alexander Ellwein

**Affiliations:** 1 Department of Orthopedics and Traumatology, Diakovere Friederikenstift, Humboldtstraße 5, 30169 Hanover, Germany; 2 Diakovere Annastift, Department of Orthopedic Surgery, Medical School Hanover, Hanover, Germany

**Keywords:** AO classification, Dubberley sub classification, Lateral approach, Headless compression screws, Double plate constructions, Total elbow arthroplasty, AO Klasssifikation, Dubberley Subklassifikation, Erweiterter lateraler Zugang, Doppelplattenosteosynthese, Kopflose Doppelgewindekompressionsschraube, Ellenbogenprothese

## Abstract

Around one third of humeral fractures and 2–6% of all fractures occur to the distal part of the humerus. There is a bimodal distribution differentiating between young male patients with high-energy and elderly female patients with low-energy trauma related to osteoporosis. The AO classification and Dubberley subclassification are used in daily routine. Most fractures are diagnosed on radiographs. For further evaluation, three-dimensional computed tomography is recommended, especially for comminuted or complex fractures. Owing to the long immobilization and resultant poor functional outcome, conservative treatment is followed for inoperable patients. The operative approach and osteosynthesis depend on the fracture pattern. In A1 avulsion fractures, open reduction and screw fixation are recommended. In A2/A3 fractures, a triceps-sparing approach following a 90° double-plate construction (radial dorsal/ulnar lateral) with locking plates is favored. Partial articular B1/B2 fractures are exposed via a medial or lateral approach using unilateral locking plates to stabilize the medial/lateral column. Coronal shear fractures (B3) are classified after Dubberley and are treated via an extended Kocher approach and headless compression screws in anteroposterior direction. If there is a further posterior comminution or a lateral column fragment, stabilization is needed for the lateral/medial column with a precontoured locking plate. In solely articular fracture patterns, a dorsal approach with either a 90° or 180° double-plate construction is advised. If a reconstruction is not possible owing to fracture complexity or bone quality, total elbow arthroplasty is a viable option. However, lifelong limitation in weight-bearing up to 5 kg, limited longevity, and the potential for complicated revision surgery should be considered.

## Zusammenfassung

Die distale Humerusfraktur stellt ein Drittel aller Humerusfrakturen und 2–6 % aller Frakturen dar. Dabei sind primär 2 Patientenkollektive zu unterscheiden: Männer zwischen 12–19 Jahren durch Hochrasanztraumata und Frauen über 80 Jahre mit osteoporotischer Knochenstruktur durch Niedrigenergietraumata. Im klinischen Alltag hat sich neben der AO-Klassifikation die Subklassifikation von frontalen Abscherfrakturen nach Dubberley als praktikabel erwiesen. Bereits im konventionellen Röntgenbild lässt sich ein Großteil der Frakturen erkennen. Zur exakten Klassifikation und Therapieplanung hat sich die Computertomographie mit dreidimensionaler Rekonstruktion insbesondere bei mehrfragmentären und komplexen Frakturen bewährt. Aufgrund längerer Immobilisation und häufig hieraus resultierenden deutlichen funktionellen Einbußen bleibt die konservative Therapie inoperablen Patienten vorbehalten. Der operative Zugangsweg und das jeweilige Osteosyntheseverfahren sind frakturspezifisch zu wählen. Dabei haben sich neben der mono-/bikondylären winkelstabilen Plattenosteosynthese die Schraubenosteosynthese und neuerdings kopflose Kompressionsschrauben mit Doppelgewinde durchgesetzt. Epikondyläre A1-Frakturen werden mittels Schraubenosteosynthese versorgt. Für metaphysäre A2-/A3-Frakturen wird ein den Streckapparat schonender paratrizipitaler Zugang und eine Doppelplattenosteosynthese in 90°-Technik favorisiert. Partiell artikuläre B1- und B2-Frakturen können entsprechend ihrer Lage entweder über einen radialen oder medialen Zugang erreicht und mittels einseitiger winkelstabiler Plattenosteosynthese versorgt werden. Frontale Abscherfrakturen (B3) werden mit einem erweiterten lateralen Zugang nach Kocher, mittels kopfloser Doppelgewindekompressionsschrauben und bei radialem/ulnarem Pfeiler mit additiver winkelstabiler Plattenosteosynthese fixiert. Vollständige artikuläre Frakturen (C1–C3) werden von dorsal dargestellt und je nach Wahl des Operateurs in winkelstabiler 90°-/180°-Doppelplattenosteosynthese fixiert. Ist eine Rekonstruktion unmöglich, stellt eine prothetische Versorgung des Ellenbogens eine Alternative da, wobei die lebenslange Limitierung der Belastbarkeit von 5 kg und limitierte Standzeiten sowie mögliche komplizierte Revisionsoperationen zu berücksichtigen sind.

## Epidemiology

Distal humeral fracture is a rare fracture entity that accounts for 2–6% of all fractures. One third of all humeral fractures occur in the distal segment with an incidence of 5.7/100,000 per year [1]. There is a bimodal distribution in the fracture pattern differentiating between young male patients with high-energy trauma and elderly female patients with an osteoporotic bone structure in low-energy trauma [1].

## Classification

**Fig. 1:**
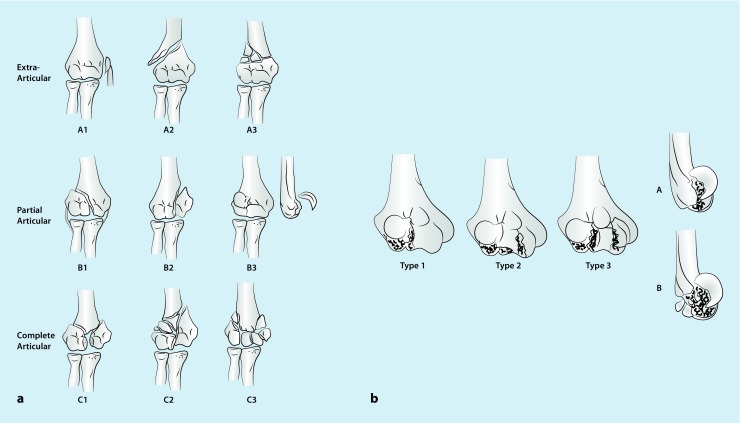
Classification of fractures of the distal humerus. **a** AO classification [5]: extra-articular (type A), partial articular (type B), and complete articular fracture (type C); **b** Dubberley classification for coronal shear fractures (AO B3 fractures; [35]): facture of the capitellum (type 1), capitellum and trochlea as single piece (type 2), and capitellum and trochlea as separate fragments (type 3) with the absence (type A) or presence of a dorsal comminution (type B)

**Fig. 2:**
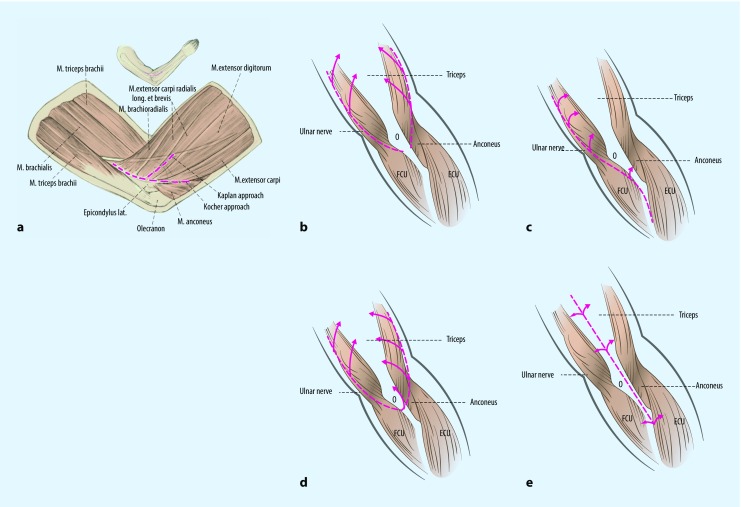
Surgical approaches: **a** lateral approach [36]; **b** paratricipital approach; **c** Bryan–Morrey approach (triceps reflecting); **d** olecranon osteotomy; **e** triceps-splitting approach. *FCU* flexor carpi ulnaris, *ECU* extensor carpi ulnaris. (**b**–**e** modified from [5])

The main characteristics of a classification system for fractures of the distal humerus are: it should be convenient for daily routine, it should assist in clinical decision-making, and it should lead to specific therapy protocols. Generally, the AO classification system is used for fractures of segment 13 and for the familiar differentiation between extra-articular, partial articular, and articular fractures (Fig. 1a; [2]).

**Fig. 3:**
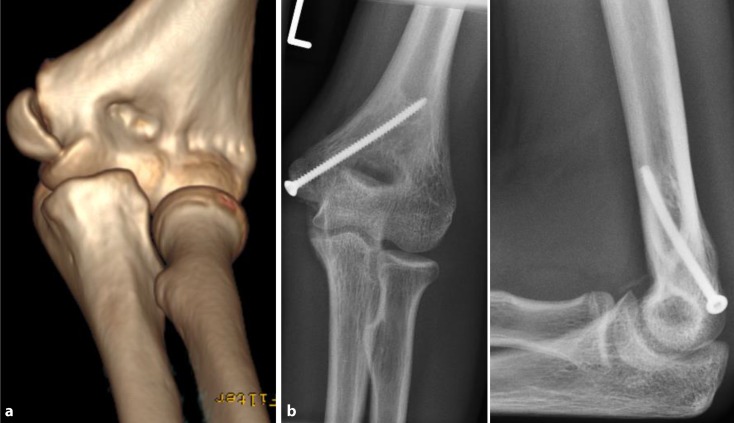
Extra-articular avulsion of the ulnar epicondyle, AO 13 A1.2 (14-year-old female patient). **a** Preoperative computed tomography (CT), **b** owing to the small fragment, the fracture is treated with only one screw

An exception is made for coronal shear fractures (AO type B3), which were further classified by Dubberley et al. in 2006 [3]. The classification describes the morphology of fractures of in the capitellum, in the trochlea, and in both areas. In addition, the classification highlights whether the posterior condyle is comminuted (Fig. 1b; [3]). In contrast to other systems, this classification directs surgical management and provides information about the outcome of the injury.

## Clinical examination

Inspecting and positioning of the injured limb are essential after a brief summary of the trauma. Most commonly, the injured elbow is in 90° flexion and supported by the contralateral side. An initial examination is necessary to detect open fractures at an early stage. An elaborate examination and documentation of the neurovascular status are mandatory, especially so as to distinguish traumatic from iatrogenic lacerations. Early conclusions about the vascular status can be made when the a. brachialis is vulnerable and the pulse of the a. radialis and the a. ulnaris is affected. Regarding the neurological status, the ulnar nerve and to a lesser degree the radial nerve are most exposed. If a fracture is assumed, unnecessary manipulation is to be avoided and the elbow is to be immobilized in a splint for further imaging.

## Imaging

Radiographs in the anterior–posterior and lateral views should be taken to determine the primary fracture. Although most fractures can be diagnosed reliably with radiographs, in multifragmentary articular or complex fracture patterns, in particular, computed tomography (CT) with three-dimensional reconstruction is mandatory. CT improves the understanding of the fracture and facilitates preoperative planning since the native radiograph is limited owing to its poor depiction of the individual fracture fragments in a complex fracture situation. Furthermore, the inter-/intraobserver reliability regarding imaging-based fracture classification is significantly higher with CT [4].

## Therapy

### Conservative therapy

Because immobilization of more than 1 week can lead to poor functional outcomes, the indications for classic conservative treatment protocols are very limited [5]. Recent conservative approaches support early mobilization while tolerating the dislocation of minor bony fragments [6]. Either way, conservative approaches are predominantly applied for low-demand geriatric patients with a high perioperative risk profile. The risks include a sixfold higher rate of pseudarthrosis and prolonged fracture healing up to four times higher than with an operative treatment protocol [1].

### Operative therapy

The aim of operative treatment is to reconstruct the articular surface anatomically and achieve stable reconstruction for early functional treatment so as to prevent limitations in elbow motion. To achieve this objective, the AO principles of an open reduction and fixation with precontoured locking plates and headless compression screws is the state of the art. Open fractures, neurovascular lacerations, or compartment syndromes require emergency surgery. All other cases are assessed as urgent and optimally treated within 24 h. Early surgical treatment improves the clinical result owing to the early functional movement and reduces the risk for complications such as infections or heterotopic ossifications [7].

## Surgical approaches to the distal humerus

### Kocher approach

The Kocher approach is a lateral approach through the musculus (m.) extensor carpi ulnaris and m. anconeus. The extended Kocher approach releases the lateral collateral ligament (LCL) and common extensor origin (CEO) to provide better exposure. In most cases, the latter is necessary for B3 fractures.

This approach is used for extra-articular fractures of the radial column (A1.1), partial articular fractures of the lateral epicondyle (B1), and coronal shear fractures (B3). The patient is in supine position with the affected arm placed on a radiolucent arm table. An incision is made proximal to the epicondylus radialis and ends distal to the radial head. The preparation is between the m. extensor carpi ulnaris and the m. anconeus. If necessary, the surgeon can extend the lateral approach to the Kocher approach lifting en bloc the LCL, the origin of the extensor muscle group, and the joint capsule (Fig. 2a; [8]).

### Medial approach

The medial approach is used mainly for extra-articular fractures of the ulnar column (A1.2), partial articular fractures of the medial epicondyle (B2), and type B3 fractures if the lateral approach is insufficient for addressing the fracture. With positioning similar to the lateral approach, a 10-cm incision is made above/under the epicondylus medialis. The second step is to view the ulnar nerve between the humeral and ulnar origin of the m. flexor carpi ulnaris. To portray the joint surface, the flexor muscle group is detached at its origin [8].

### Posterior approach

All other fractures are treated via a posterior approach. Commonly, the patient is in prone position with a small radiolucent arm table with a 90° flexed elbow. The incision starts at the distal part of the humerus, circumvents the olecranon radially, and continues on the edge of the ulna. The next step is to prepare the ulnar nerve at is exit point on the triceps [8]. Different variations in preparation—through or beside the muscle fibers—are possible so as to reach the fracture.

### Triceps-sparing approach

For the triceps-sparing approach (paratricipital approach), a window is created between the medial and lateral head of the triceps with which the triceps is mobilized from the humeral shaft. While preserving the extensor muscle group, the articular view is limited ([8]; Fig. 2b).

After the aforementioned incision, the triceps is mobilized medially for the Bryan–Morrey approach (triceps reflecting; Fig. 2c). Then the ulnar fascia and triceps are shifted en bloc radially to expose the olecranon and at the end of the surgical procedure sutured transosseously.

### Olecranon osteotomy

Olecranon osteotomy (Fig. 2d) is the approach that offers the greatest exposure of the distal humeral articular surface [9]. A V-shaped Chevron osteotomy is created with the oscillating saw and completed with a chisel [8]. The inhomogeneous fracture zone facilitates repositioning at the end of surgery. Although the articular surface is broadly exposed, the complication rate is as high as 50%. Nonunions, implant loosening, and implant irritation are some of the most common complications.

### Triceps-splitting approach

For the triceps-splitting approach, the triceps tendon is split centrally along its muscle fibers subperiosteally up to the tip of the olecranon. The surgeon should try to preserve the connection between the m. anconeus and m. flexor carpi ulnaris (Fig. 2e).

### Campbell’s approach

Campbell’s approach is a triceps-splitting approach that is an extension of the proximal ulna. The triceps muscle is detached at the proximal ulna with a periosteal radial and ulnar flap to create a wide view of the articular surface. Transosseous refixation of the triceps at the end of the surgery is mandatory [8].

## Fracture-pattern-associated approach and fixation technique

A decisive fracture classification system is required to choose the correct surgical approach and fixation technique.

### Epicondyle fracture (AO 13 A1)

Epicondyle fractures are characterized as extra-articular avulsion fractures of the ulnar or radial collateral ligaments and the muscle origin of the flexor or extensor group of the forearm. As a result of the muscle tension, secondary dislocation is inevitable and should be prevented with surgery. Depending on the epicondyle, a medial or lateral approach is used followed by open reduction and screw fixation. To secure a rotational stability, two screws are preferred (Fig. 3).

### Metaphyseal fractures (AO 13 A2/A3)

**Fig. 4:**
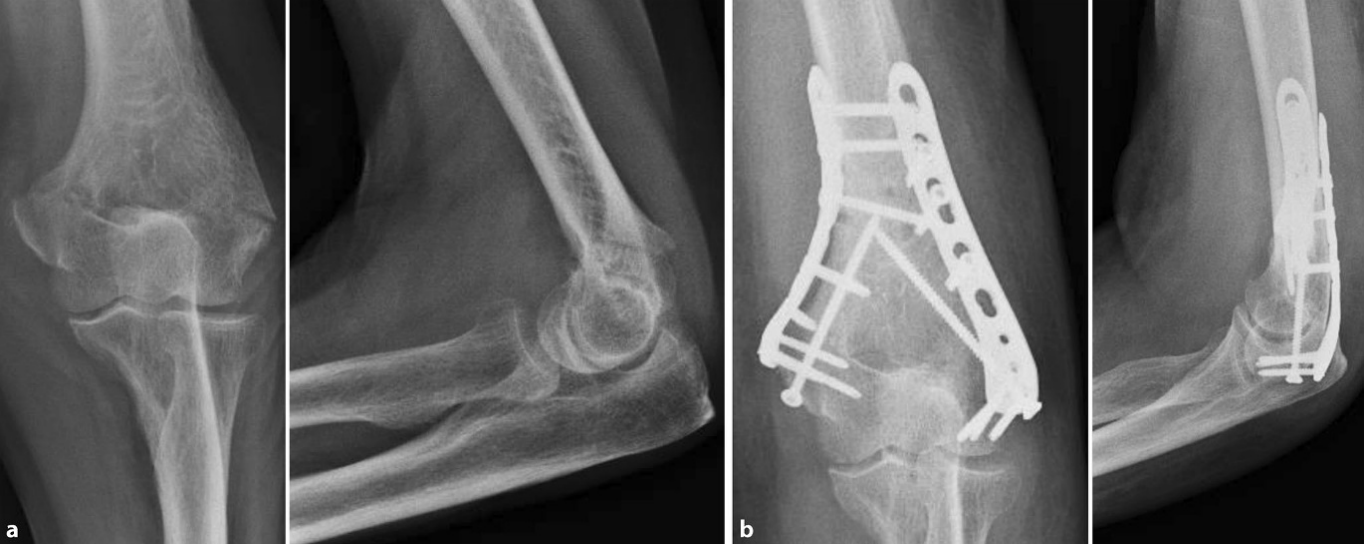
Extra-articular metaphyseal fracture, AO 13 A2 (60-year-old male patient). **a** Preoperative radiographs; **b** 90° double-plate construction (radial dorsal/ulnar) with anatomically preshaped 3.5 mm locking compression plates (LCP) and free cortical screws

Metaphyseal fractures are treated with a triceps-sparing approach followed by a 90° double-plate construction (radial dorsal/ulnar lateral) with anatomically preshaped locking plates. To gain sufficient primary stability, at least two screws should be placed bicortically proximal and distal to the fracture zone (Fig. 4).

### Partially articular fractures (AO 13 B1/B2)

Depending on the fracture position, a medial or lateral approach is suitable. In the past, surgeons often used only screws for the fixation. Owing to the aforementioned low-energy trauma in elderly patients with an osteoporotic bone structure and much-needed primary stability, this concept is now outdated. A unilateral plating to stabilize the lateral/medial column with precontoured locking plates with additional screws is recommended.

### Coronal shear fractures (AO 13 B3)

Coronal shear fractures are highly complex fracture patterns that are classified after Dubberley (Fig. 1b). Owing to the joint alignment from the distal humerus and the elbow, in addition to the oft-observed multifragmentary intra-articular fracture pattern, CT with three-dimensional reconstruction is essential.

**Fig. 5:**
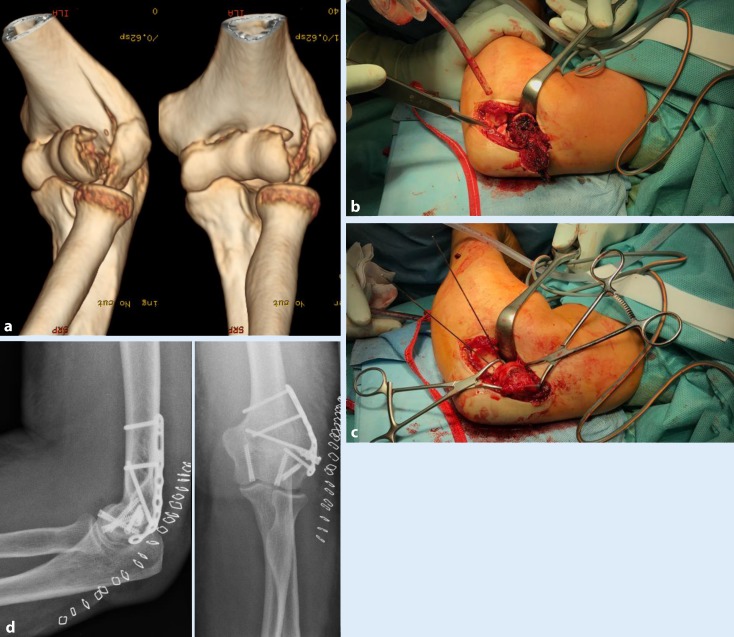
Coronal shear fracture with radial metaphyseal fracture extension, AO 13 B3/Dubberley 2B (54-year-old female patient). **a** Preoperative computed tomography image with three-dimensional reconstruction; **b** extended radial approach with surgical radial dislocation; **c** reduction and temporary fixation with K‑wires; **d** headless compression screws in anterior–posterior direction and an additive locking plate

A radial or extended radial approach is used to assess coronal shear fractures. After visualizing the fracture, one has to distinguish between solely coronal shear fracture, multifragmentary coronal shear fractures, and coronal shear fractures with or without the presence of a posterior comminution (Dubberley subclassification type A/B, Fig. 1b). Solely coronal shear fractures can be assessed with headless compression screws in anterior–posterior direction (Fig. 5; [10]), which show improved biomechanical stability than in posterior–anterior direction [10]. Often, AO B3 fractures have either a multifragmentary aspect or a posterior comminution. For these fractures, the lateral/medial column is primarily stabilized with a precontoured locking plate [3]. After this step, temporary fixation of the capitellum/trochlea is performed with K‑wires [3]. When anatomic reduction is achieved, the osteosynthesis is finalized by using headless compression screws. While closing the wound, it is critical to ensure that—depending on the approach—the LCL/CEO and/or the ulnar collateral ligament (UCL)/common flexor origin (CFO) complex are reattached.

### Articular fractures (AO 13 C1–C3)

Independent of age and gender, current thinking when assessing C1–C3 fractures is still to reduce the fracture anatomically and perform a stable osteosynthesis. Anatomically precontoured angular stable plates are commonly used. It remains controversial whether a 90° or 180° double-plate constructions is superior [11]. There are biomechanical benefits to the 180° construction [12], but clinically no difference is seen. In our opinion, the plate positioning should be decided by the surgeon according to the fracture morphology.

Besides the construction, an antiparallel plate positioning was reported to be biomechanically superior in preventing peri-implant failure [13]. Recent biomechanical studies refute this belief and highlight the importance of the proximal screws. Proximal screw positioning at different levels is seen as the major factor in avoiding peri-implant failure [14]. As many screws as possible should be placed in the distal metaphyseal fracture fragment to enhance stability.

**Fig. 6:**
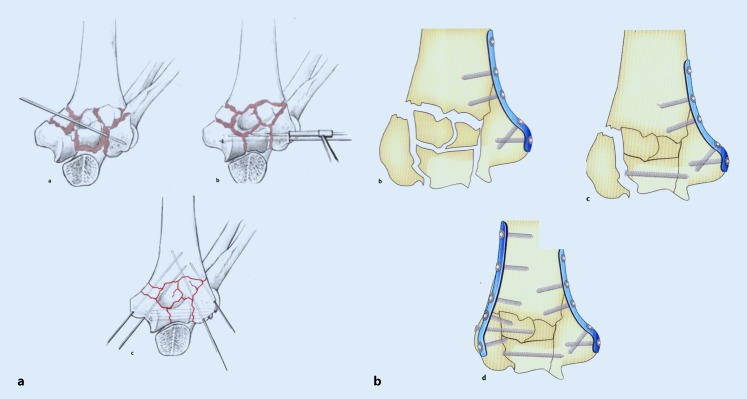
Repositioning techniques for complete intra-articular fractures. **a** Repositioning of the joint unit, transcondylar screw, and repositioning against the shaft; **b** column reposition: reconstruction of one column and repositioning of the joint unit against the column

Generally, the fracture is exposed via a dorsal approach. One strategy is to reduce the articular block provisionally with K‑wires and in a second step position the block correctly to the humeral shaft followed by the bicondylar plate osteosynthesis (Fig. 6a).

When the fracture presents a radial or ulnar column it should be addressed primarily. After the repositioning and fixation of the first column with a common angular stable locking plate osteosynthesis, further fragments can be arranged around it. The olecranon groove serves as a guide for further fragments. Finally, the second column should be fixed with another angular stable locking plate (Fig. 6b).

## Arthroplasty for distal humeral fractures

### Hemiarthroplasty

Depending on the fracture pattern, it may be impossible to make a rigid construct allowing for early full range of motion [15]. In the case of a multifragmentary fracture type with a severely comminuted articular surface, low-plane fractures, or poor bone quality caused by osteoporosis or arthritis, arthroplasty can be considered [15]. Similar to other joints, hemiarthroplasty can be considered in distal humeral fractures.

Moreover, in young, active patients, hemiarthroplasty is reported to be more durable with less stringent limitations compared with total elbow arthroplasty [16]. This is explained by less implant loosening and polyethylene wear due to the lack of an ulnar component and elimination of the polyethylene [16]. However, the indications for hemiarthroplasty are greatly limited by anatomical requirements. It is mandatory to have a stable elbow joint with intact or reconstructable radial head, coronoid, and ulna in addition to intact or reconstructable collateral ligaments [15–17]. Therefore, the advantage of the reduced operative time mentioned in the literature is counteracted by the simple fact that the collateral ligaments are affected to a corresponding degree, leading to complex reconstruction with the challenge of correct tension [16, 17]; especially since the unlinked nature of the hemiarthroplasty requires mandatory correct placement for stable kinematics [15]. Furthermore, mid- and long-term results are still missing and the impact of the implant on the ulnar and radial cartilage is unknown.

In conclusion, there is a narrow range of indications for hemiarthroplasty, involving a steep learning curve. We acknowledge the role of hemiarthroplasty, but to date we do not advocate hemiarthroplasty because of the aforementioned reasons.

### Total elbow arthroplasty

**Fig. 7:**
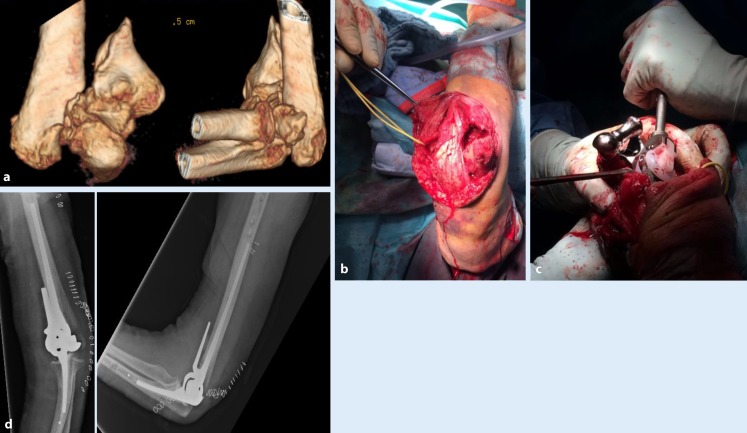
Nonreconstructable distal humeral fracture, AO 13 C3 (88-year-old female patient). **a** Preoperative computed tomography with three-dimensional reconstruction; **b, c** implantation of cemented linked total elbow arthroplasty using a triceps-on approach; **d** postoperative radiographs

Historically used in cases of severe rheumatoid arthritis, recent advances in implant materials have made total elbow arthroplasty a viable option in nonreconstructable distal humeral fractures ([18]; Fig. 7).

Generally, one can differentiate between linked models, which contain a linkage between the humeral and ulnar components, and unlinked models [15]. In the past, most linked total elbow arthroplasties (TEA) where constrained models with simple hinges that led to an excessive amount of shear stress with subsequent high rates of aseptic loosening and wear on the material and thus ultimate failure [15]. Modern TEA are semiconstrained with a sloppy hinge allowing for ±7° varus/valgus motion [19]. Furthermore, current TEA have an extracortical anterior flange, which improves rotatory and posterior stability and is thought to reduce humeral component loosening thanks to a superior load transfer [15]. To achieve stable fixation, the humeral component as well as the ulnar component are cemented [19]. If necessary, modern modular models allow for additive radial head arthroplasty [15].

After a typical dorsal incision slightly radial to the tip of the olecranon, the authors recommend a triceps-preserving approach like the triceps-on approach [15, 19]. The advantage of this procedure is an early functional range of motion and less postoperative triceps weakness [15]. With TEA, most patients have sufficient pain relief and an enhanced range of motion. However, in most cases a minor deficiency in extension remains postoperatively and should be addressed in the preoperative clarifications to patients [19]. Further complications include infection (3.3%), ulnar nerve dysfunction (3%), aseptic loosening, triceps weakness or rupture, and mechanical complications [15, 19].

In conclusion, TEA requires a precise indication and its main benefit should be viewed in the context of pain relief and an acceptable to good postoperative range of motion. In view of the lifelong activity restrictions with a limited weight-bearing of 5 kg, limited implant survival, and limited revision options, reconstruction should be the treatment of choice.

## Fracture management in the elderly

The aforementioned therapy concepts are fully applicable to the elderly population. Premature presumptions of what the patient’s desires may be concerning osteosynthesis or elbow joint replacement without aged-based decision-making are risky. Newer studies report excellent results for precontoured angular stable locking plate osteosynthesis [20–22]. The main reasons for failure are loss of reduction and secondary dislocation of implant material. Postoperative immobilization can be an option in exceptional cases to protect the osteosynthesis. If reconstruction is not possible because of fracture complexity or bone quality, recent studies have shown excellent functional outcome, low failure rates, and entirely pain-free outcomes for total elbow joint replacement [18, 23]. The disadvantages are the life-long limitation in weight-bearing of up to 5 kg, the limited longevity of the implant, and the potential for complicated revision surgery [24].

Thus, the decision between a primary osteosynthesis or joint replacement in the elderly should be made on an individualized basis and should take into account fracture complexity, bone quality, and the patient’s personal goals.

## Complications

The complication rate for fractures of the distal humerus is up to 30% and complications fall within one of three major categories [25]: primary-trauma-associated complications, intraoperative complications, and postoperative complications.

In general, the elbow is characterized by a small intracapsular volume, leading to effusion, scarring, hemarthrosis, and ultimately thickening of the capsule. Owing to these anatomical characteristics, stiff elbow is a common occurrence after trauma [26].

According to Morrey et al., the “functional arc” for proceeding with 90% of daily activities is in extension/flexion 0–30–130° and in pronation and supination 50–0–50° [27]. There are intrinsic factors involved such as osteophytes, adhesion, and joint alignment and extrinsic factors such as heterotopic ossification, capsular adhesions, and muscular contractions. After surgical procedures on a distal humeral fracture, there is a 14% rate of heterotopic ossification [28]. In type C fractures, the prevalence of heterotopic ossification rises to 26%. Another dominating risk factor is delayed surgical treatment. To prevent heterotopic ossification, two main strategies are employed: administration of indometacin after surgery or single-dose one-time radiotherapy performed postoperatively with 7 Gy [29]. The success rates are low in the general population but some benefits have been reported for high-risk patients [5, 29]. Therefore, clear evidence is missing. Nevertheless, our treatment concept for every patient with a type C fracture is to prescribe 50 mg indometacin (1–0–1) for 14 days [30, 31].

### Comminuted injuries

The neurovascular anatomy in the elbow joint after fracture is complex and depends on the fracture pattern. From the vascular point of view, the brachial artery, which reaches the elbow through the cubital fossa and divides into the radial and ulnar artery, is at risk for injury. Because of the high number of collateral branches, the radial and ulnar pulse is not clearly discernible and if in doubt further evaluation is required. In these cases, Doppler ultrasonography, if necessary angiography, and consultation with a vascular surgeon are imperative [32].

### Neural damage

While posttrauma-associated injuries mainly concern the radial nerve, the ulnar nerve is most at risk for iatrogenic injury. To prevent iatrogenic damage, we recommend visualizing the ulnar nerve and securing it with a vessel loop. In this context, ventral transposition has been described in the literature. It has been reported that, in trauma following injuries to the ulnar nerve, ventral transpositions have positive outcomes [33]. In cases where there is no irritation of the ulnar nerve by the implants, a routine ventral transposition has no advantages [5].

### Pseudarthrosis

Usually, there is a nonunion rate of 2–10% after a distal humeral fracture. Insufficient osteosynthesis and secondary loss of reduction are caused primarily by material failure. The main aim here is to restore the joint surface after addressing the pseudarthrosis region with autologous bone graft and an appropriate re-osteosynthesis.

### Infections after fracture

Postoperative infections occur in up to 12% of cases depending on the initial soft-tissue damage and the time from trauma to surgery [34]. Delayed surgery is associated with a significantly increased risk of infection. Obvious clinical signs are rubor, tumor, calor, dolor, functio laesa. In the case of an infection, general therapy concepts are followed until implant removal.

## Postoperative care

The main objective of a surgical procedure is early mobilization with an unrestricted range of motion to prevent elbow stiffness. In our therapeutic approach, patients receive a pain catheter preoperatively that enhances the physical therapist-assisted exercises from day 2 after surgery. Any weight-bearing is prohibited for the first 6 weeks after surgery. In the past, owing to morbidity related to the approach, we limited flexion to 90° for 6 weeks after elbow arthroplasty. When using triceps-preserving approaches such as the triceps-on approach, a free range of motion can be achieved even after arthroplasty.

If there is any doubt with the findings on postoperative radiographs, CT with three-dimensional reconstruction is undertaken to perform an early revision if needed.

## Conclusion

Distal humeral fracture is a rare and highly complex entity. It is mandatory to employ an accurate fracture classification system from which to derive a fracture-pattern-associated approach and fixation technique. In multifragmentary or uncertain fracture patterns, CT with three-dimensional reconstruction is obligatory. Because long-term immobilization of the elbow almost always leads to elbow stiffness, conservative treatment protocols only play a role for inoperable geriatric patients. The state of the art is anatomical reduction and fixation either with conventional screws, headless compression screws, or precontoured uni-/bicondylar locking plates. In nonreconstructable fracture situations, total elbow arthroplasty is a viable therapy option, considering a life-long limitation in activity and weight-bearing in the affected limb, limited implant survival, and restricted revision options.
